# Dimensions of Entrepreneurial Success: A Multilevel Study on Stakeholders of Micro-Enterprises

**DOI:** 10.3389/fpsyg.2018.00791

**Published:** 2018-05-28

**Authors:** Wiktor Razmus, Mariola Laguna

**Affiliations:** Institute of Psychology, The John Paul II Catholic University of Lublin, Lublin, Poland

**Keywords:** entrepreneurial success, entrepreneurs, stakeholders, multilevel analysis, factor analysis

## Abstract

The study provides an insight into the indicators and dimensions of entrepreneurial success as evaluated from the external stockholders’ perspective. As each firm is embedded in a network of relations with stakeholders (business partners), understanding how they evaluate entrepreneurial success is important. The initial qualitative study in the form of in-depth interviews allowed us to identify the indicators of entrepreneurial success that are identified by external stakeholders of micro-firms. In the quantitative study on 475 stakeholders of 57 micro-firms, we identified the dimensions of entrepreneurial success. Using a multilevel approach, we found six dimensions of entrepreneurial success at the individual stakeholder level and four dimensions at the firm level. The results show that stakeholders perceive entrepreneurial success in terms of many dimensions, not focusing solely on economic indicators. This knowledge may inform micro-firm management and the strategies employed by practitioners supporting entrepreneurs.

## Introduction

Success in running a company is of vital importance not only for entrepreneurs-business owners themselves but also for the society as a whole, since it leads to economic growth and job creation (e.g., van Praag and Versloot, 2007). Two kinds of indicators based on which businesses are judged to be successful have been considered in past research: financial indicators of firm performance (e.g., McGee et al., 1995; Murphy et al., 1996; Zhou et al., 2017) and indicators of entrepreneur’s satisfaction from running a firm (e.g., Gorgievski et al., 2011; Fodor and Pintea, 2017). Success evaluations were usually based on assessments made by entrepreneurs themselves (Dijkhuizen et al., 2016; Przepiorka, 2016; Wach et al., 2016). Indicators of business success as evaluated by entrepreneurs, however, may be different from those taken into account by people who judge firms from the outside, as was observed in early research (Montagno et al., 1986). Still, our knowledge of what kind of indicators and dimensions of entrepreneurial success are of importance to stakeholders (e.g., suppliers, consumers, or retailers) is very limited.

In order to fill this gap, the present study provides an insight into entrepreneurial success from stockholders’ point of view. We recognize that entrepreneurs are embedded in a larger social context, which includes relations with their stakeholders and business partners. If stakeholders are not satisfied with the performance of a firm, they withdraw their support, which may threaten the survival of the business (Ghosh et al., 2001). Moreover, there has been a longstanding call for investigating entrepreneurship from a multilevel perspective, taking into account the direct environment of businesses (Low and MacMillan, 1988). Our study answers this call by investigating entrepreneurial success dimensions at two levels of analysis based on stockholders’ evaluations.

We investigate what dimensions stockholders use to evaluate the entrepreneurial success of micro-enterprises. We focus on this kind of firms for two reasons. First, small and micro-enterprises constitute the overwhelming majority of enterprises in the United States (US Census Bureau, 2012) and in the EU (Eurostat, 2015), and they provide the majority of private employment (Observatory of European SMEs, 2007). Second, in micro-firms employing no more than 10 people there are direct relationships between entrepreneurs and their stakeholders, which allows stakeholders to observe and evaluate not only the financial performance of firms but also many other indicators of entrepreneurial success which may be unnoticeable when cooperating with bigger companies.

After explaining our conceptual framework in more detail, we present a multilevel quantitative study. This study was based on the results of the initial qualitative study using in-depth interviews, which allowed us to identify the kinds of indicators used by stakeholders when talking about the success of their business partners.

### Evaluations of Entrepreneurial Success

Popular opinion has it and many studies show (Baron and Markman, 2003; Crane and Sohl, 2004; Steffens et al., 2009; Unger et al., 2009) that entrepreneurial success is evaluated from a financial perspective and identified with the financial yield of the company (Steffens et al., 2009; Zhou et al., 2017). High financial outcomes and good position on the market are named as success indicators, together with other economic indicators of entrepreneurial success and firm performance (van Praag and Versloot, 2007; Richard et al., 2009). Company success is also often identified with firm’s growth, meaning an increase in financial outcome, productivity, and the number of employees (Brandstätter, 2011). One of the common criteria of entrepreneurial success defined in this way is sales growth (Florin et al., 2003; Zhou et al., 2007; Steffens et al., 2009; Achtenhagen et al., 2010). However, empirical studies (e.g., Kiviluoto, 2013) and theoretical analyses (e.g., Rauch and Frese, 2000) show that basing assessment of entrepreneurial success solely on economic indicators limits our understanding of this phenomenon. It is crucial to look for other indicators especially when the success of micro-firms is considered (Davidsson, 1989; Greenbank, 2001), since financial indicators of small and micro-firms do not always adequately reflect their performance (Walker and Brown, 2004; Reijonen and Komppula, 2007). Therefore, as regards big enterprises, the economic approach can serve as the basis for defining their success on the market, but this is not the case with micro- and small firms (Greenbank, 2001). Many micro-enterprises are doing well, even though their profit is not vast or increasing and even though the number of their employees does not increase. A study conducted on a sample of 400 entrepreneurs running micro and small firms showed that 40% of them did not set company growth as the goal (Davidsson, 1989). This proves that the economic approach is insufficient to define such a complex phenomenon as the entrepreneurial success (Walker and Brown, 2004). Therefore, deeper insight into the indicators of entrepreneurial success is needed, and the psychological approach may broaden our understanding of this issue.

What is stressed in the psychological approach is the need to assess entrepreneurial success using subjective evaluation criteria, applied by entrepreneurs themselves (Wach et al., 2016). Previous research has shown that such subjective evaluation of success covers a wide range of indicators, including perceived attainment of valuable goals (Buttner and Moore, 1997; Walker and Brown, 2004), personal satisfaction (Fisher et al., 2014), work-life balance (Buttner and Moore, 1997), and satisfaction with business performance (Gorgievski et al., 2014). An extensive analysis of the literature on management, business, entrepreneurship, and psychology done by Gorgievski et al. (2011) provides a categorization of entrepreneurial success criteria as seen from the business owner’s perspective. Apart from the traditional economic indicators, such as profitability and growth (e.g., increase in the number of employees or in sales), the authors took into account some less obvious indicators such as innovation (e.g., introduction of new products or methods), firm survival/continuity, contributing back to society (e.g., socially conscious and sustainable production methods). More subjective criteria of entrepreneurial success were also listed in this classification, such as personal satisfaction, work-life balance related to having time for family, friends, and leisure activities. Important success indicators concerned functioning in external environment: stakeholder satisfaction, public recognition and good reputation, as well as social utility (e.g., fulfillment of some society needs). This shows that, beside financial profit and company growth, entrepreneurs use many other criteria when evaluating their entrepreneurial success. Assessing the quality of the functioning of their firms, they also appreciate the significance of relations with the broader environment.

Former analyses and studies of entrepreneurial success using this kind of subjective rather than only economic indicators (Buttner and Moore, 1997; Walker and Brown, 2004; Gorgievski et al., 2011, 2014; Fisher et al., 2014; Fodor and Pintea, 2017) focused on the entrepreneurs’ perspective, however. The open question remains how the external environment, such as stakeholders, perceives entrepreneurial success. One of recent qualitative studies (Kiviluoto, 2013) suggested that purely economic indicators (e.g., sales growth) are not sufficient to evaluate entrepreneurial success from the stakeholders’ perspective. Therefore, there is a need for a more holistic approach when looking for a better understanding of the complexity of firm success (Kiviluoto, 2013).

### Entrepreneurial Success From the Stakeholders’ Perspective

Entrepreneurs do not act in a vacuum but constantly operate in a social environment, which they remain in mutual relations with. Their firms are embedded in relationships with many stakeholders. Stakeholders are all individuals, groups, and other organizations who have an interest in the actions of an organization and who have the ability to influence it (Savage et al., 1991). Two groups of stakeholders are distinguished: external stakeholders (customers, suppliers, competitors, financial institutions) and internal stakeholders (shareholders, employees), who control the company’s activity by virtue of ownership or position in the company (Andruszkiewicz et al., 2014). The group that we are interested in is external stakeholders. Following the strategic management theory (Freeman, 1984), which states that companies must be managed in accordance with the interests of their stakeholders, different strategies of managing relationships with external partners are discussed in the management literature (e.g., Freeman, 1984; Savage et al., 1991). There is agreement, however, that not only big companies but also micro-enterprises shall take it into account that if stakeholders are not satisfied with the effects of the cooperation and do not perceive the firm as a successful partner, offering stable collaboration, they may withdraw their support. This, in turn, may diminish the firm’s business performance or even threaten its survival (Ghosh et al., 2001).

However, firms not only operate in the social milieu themselves but are also perceived by external partners. For example, early research (Montagno et al., 1986) showed that there were significant differences in the dimensions contributing to entrepreneurial success as viewed by different groups. Taking this into account, we can distinguish internal and external perception of entrepreneurial success. Despite the fact that entrepreneurial success has been a widely studied issue (Davidsson, 1989; Buttner and Moore, 1997; Greenbank, 2001; Walker and Brown, 2004; Fisher et al., 2014; Gorgievski et al., 2014), previous studies have been mostly limited to the internal perspective. They concentrated on the firm itself and evaluated its success using mainly entrepreneurs’ appraisals. Thus they left aside the important question of how entrepreneurial success is evaluated by the external social environment. Due to the commonly observed fact that the success of any business depends on close and fruitful cooperation with external partners (e.g., Freeman, 1984; Savage et al., 1991), the investigation of this external perspective is very important.

The current study aims at filling this gap. Its objective is to explore what dimensions are used for the evaluation of entrepreneurial success by the broad social environment – by stakeholders of micro-firms. A better understanding of the external evaluation of success, its indicators and dimensions, may help to identify what is appreciated by the environment. This, in turn, may help micro-entrepreneurs manage the social image of their firms. As each firm has many stakeholders (e.g., suppliers, consumers, retailers), the picture of external perception of entrepreneurial success shall take this into account. And so, we can distinguish two levels: the individual person level, i.e., the evaluation of success made by an individual stakeholder, and the firm level, i.e., the evaluation of success of the whole firm made by all of its stakeholders. We shall analyze these two levels simultaneously to provide a comprehensive understanding of external evaluation of entrepreneurial success. Due to the scarcity of research on entrepreneurial success taking into account the perspective of external stakeholders, our study is of exploratory nature. Therefore, we do not formulate any hypothesis but only pose a research question:

*Research question*: What are the dimensions of entrepreneurial success evaluated by the external stakeholders of micro-firms?

## Materials and Methods

### Initial Qualitative Investigation of Success Indicators

The aim of this initial study was to find out what indicators of entrepreneurial success are listed by external stakeholders of micro-firms. We then used these indicators to develop statements describing entrepreneurial success from an external perspective (see section “Measure” below).

#### Participants

The stakeholders who were invited to take part in an in-depth interview met two criteria: (a) cooperating with micro-firms as external partners (b) for at least a year. The participants in the study were 12 suppliers and customers, including five women (age *M* = 36.91, *SD* = 9.34). Nine of them had higher education and three had secondary education.

#### Research Tool and Procedure

We conducted in-depth interviews. The interview consisted of two parts. First, respondents were asked to answer open questions: *What do you understand by “company’s success”? Please think of one or a few companies that you know from your surroundings which, in your opinion, have achieved success. What makes a company successful?* In the second part, respondents were asked to what extent, in their opinion, the categories of success indicators listed below were meaningful for entrepreneurial success. If an indicator was listed in the first part of the interview, it was not elicited again. Interviews were conducted by trained researchers at the respondents’ homes or workplaces. After consent had been obtained from the respondent, the interview was audio recorded. We took into account 11 categories of entrepreneurial success indicators, which had been extracted from earlier studies and analyses (Buttner and Moore, 1997; Walker and Brown, 2004; Gorgievski et al., 2011): (1) firm survival/continuity (survival on a market); (2) profitability of the firm (e.g., revenue, share of the market, good profit margin); (3) growth of the firm (e.g., increase in the number of employees, market share, and/or distribution); (4) image of the firm (e.g., good reputation, positive image of the firm); (5) clients satisfaction (e.g., having regular customers); (6) social responsibility (e.g., taking part in social campaigns, helping local communities); (7) innovation of the firm (e.g., new services/products, investing in new solutions); (8) implementation of the business plans (e.g., reaching set goals, bringing to life the vision and mission of a company); (9) entrepreneur satisfaction (e.g., job satisfaction, treating work as a calling); (10) entrepreneur work-life balance (e.g., not bringing work-related emotions home, having time for family and other activities); (11) employees satisfaction (e.g., employees’ engagement in the firm, lack of turn-over intention).

#### Data Analysis Strategy

Participants’ responses were transcribed and divided into “meaning units” – fragments of text that had an identifiable theme. Next, five competent judges assigned respective units to one of 11 categories of entrepreneurial success indicators (0 – *does not belong to this category*; 1 – *belongs to this category*). A unit was assigned to a given category when at least three out of five judges agreed about it. All judges had been trained on coding and performed a trial coding of 100 units. Agreement among judges was assessed using Krippendorff’s alpha coefficient, whose values range from 0 to 1; the closer to 1 they are, the higher the inter-rater agreement (Hayes and Krippendorff, 2007). Next, we analyzed the frequency of the entrepreneurial success indicators.

#### Results

We obtained 502 meaning units describing indicators of entrepreneurial success. In order to verify the reliability of categorization of these units, we conducted the analysis of inter-rater agreement. Krippendorff’s alpha values ranged from 0.47 for the category called implementation of the business plans to 0.80 for firm survival/continuity (**Table 1**). This shows relatively high agreement between judges, proving assurance in the validity of research results (Hayes and Krippendorff, 2007), which made it possible to meaningfully analyze the frequency of the respective indicators of entrepreneurial success (**Table 1**). This analysis showed that all of the categories of entrepreneurial success were mentioned by stakeholders in the interviews. The most frequently listed categories were image of the firm, profitability of the firm, and employees satisfaction, whereas the least frequent ones were entrepreneur work-life balance and entrepreneur satisfaction. Indicators of entrepreneurial success listed by stakeholders in this qualitative study were next used as a base for development of statements for a quantitative study described below.

**Table 1:** Indicators of entrepreneurial success from interviews with external stakeholders.

Category of indicators	Krippendorff’s α	Percent of responses	Examples of statements
Firm survival/continuity	0.80	3.3	‘Firm is on the market long enough to be successful’; ‘firm is on the market for many years’
Profitability of the firm	0.67	9.2	‘Firm makes profit’; ‘economic success’
Growth of the firm	0.63	6.7	‘Firm does not stand still’; ‘the whole time firm is expanding the scope of activity’
Image of the firm	0.67	10.7	‘Image of the firm is probably the most important indicator’; ‘the way a firm is seen by partners’
Clients satisfaction	0.72	6.7	‘Firm knows the expectations of customers’; ‘recommending the firm to others’
Social responsibility	0.77	4.0	‘Whether firm is active in social life’; ‘not using chemicals which weakens the plant’
Innovation of the firm	0.78	5.6	‘New machines make the job easier’; ‘new way of building relations’
Implementation of the business plans	0.47	3.4	‘Reaching set goals’; ‘determining priorities’
Entrepreneur satisfaction	0.54	2.7	‘Entrepreneur is personally happy about his work’; ‘personal success’
Entrepreneur work-life balance	0.59	2.5	‘Big success to have time for work and for home’; ‘well-organized work time’
Employees satisfaction	0.71	8.6	‘Employees are happy about their salaries’; ‘employer knows, that staff is content about the work’


### Procedure

Data collection consisted of two stages. First, trained researchers contacted entrepreneurs, and after obtaining their informed consent they asked each entrepreneur to provide the contact data of eight to 10 of their business partners. In the second stage, researchers contacted these business partners directly or asked entrepreneurs for help in collecting the surveys completed by their business partners. Paper-and-pencil questionnaires were collected anonymously in sealed envelopes, to ensure the confidentiality and anonymity of the data. Each of the business partners evaluated the success of a given company, whose name was provided in the heading of the questionnaire.

The entrepreneurs invited to take part in this study met (conjointly) five criteria: being (a) the founder, (b) the manager, and (c) the owner or co-owner; of a firm that (d) hired from one to 10 employees (excluding the entrepreneur), and (e) had existed on the market for at least 2 years. Business partners met two criteria: (a) they had collaborated with a given firm (b) for at least 1 month.

### Participants

Out of 131 entrepreneurs invited to take part in the study, 72 declined; questionnaires from two firms were excluded due to incomplete data from business partners. Finally, 57 micro-enterprises (43.5% of the initially invited sample) took part in the study; the entrepreneurs (including 23 women) provided the contact data of the stakeholders of their firms. The firms run by the entrepreneurs had been present on the market from two to 27 years (14 years on average) and hired from one to 10 employees (*M* = 4.04, *SD* = 3.67). They operated in the service sector (61.4%), trade (45.6 %), production (10.2%), construction (10.5%), craft (5.3), and other sectors (1.8%); some firms operated in more than one sector simultaneously.

A total of 590 business partners were invited to take part in the study; 475 of them participated in it (80.5% of the initial sample). There were from six to 10 stakeholders (*M* = 8.35, *SD* = 0.94) per firm. The participants were 313 men and 162 women, aged 18–70 (*M* = 38.58, *SD* = 10.10). As regards their education, 50.5% had obtained a university diploma, 31.6% had finished secondary school, and 14.5% had finished vocational school. Most business partners (52.2%) represented the trade sector, 41.3% operated in the service sector, 9.6% were from the production sector, 5.7% represented the construction sector, 1.3% operated in the craft sector and 3% were from other sectors (some participants represented firms active in a few sectors). They were suppliers (53.2%), buyers (35.3%), or intermediaries (4.5%) of the micro-firms. The average time of cooperation was more than 5 years (from one to 312 months; *M* = 65.08 months).

### Measure

Based on the results of the in-depth interviews conducted during the initial qualitative study, statements describing entrepreneurial success were generated. Their linguistic correctness and comprehensibility were verified by two independent linguists and 10 employees (including four women) working in different enterprises, and the content of some statements was corrected accordingly. In this way, 57 statements describing entrepreneurial success were generated. They were preceded by the following instruction: “Please rate the extent to which the following statements are true about the company you cooperate with, using a scale from 1 – *not true at all* to 5 – *very true.*”

### Data Analysis Strategy

The aim of our analyses was to determine the factorial structure of entrepreneurial success as evaluated by external stakeholders. As each firm has many external stakeholders, our data has a natural multilevel structure. The first level of this structure (i.e., Level 1) is the stakeholder level – evaluations of success made by individual stakeholders. This first level is nested within a second level (i.e., Level 2), which is the firm level. This nested structure of data (Nezlek, 2012) makes it possible to capture both the fact that each firm has different stakeholders who evaluate its success differently (which is captured at Level 1) and the fact that success evaluations differ also across firms (which is captured at Level 2). We took this into account in two stages of data analysis.

In the first stage, we performed the initial exploratory factor analysis (EFA) on data that were centered around the group mean (Nezlek, 2012), i.e., around the mean evaluation of entrepreneurial success of all external stakeholders of each firm. The values calculated in this way reflect the evaluation of success as a gap between the assessment done by each stakeholder and the average evaluation of a given firm. We performed the EFA using principal component analysis with Oblimin rotation (delta = 0) and Kaiser normalization.

The aim of the second stage of analysis was to verify the factorial structure of entrepreneurial success at two levels simultaneously. The estimation of a single-level model in the presence of nested data structure may generate underestimated variances and standard errors (Bryne, 2012). To control this, we used the multilevel approach, allowing for the partition of the total variance into components at the stakeholder level (Level 1, the individual-level data purged of firm-level variability) and the firm level (Level 2). We performed the two-level EFA with Oblimin rotation and maximum likelihood (ML) estimation on non-centered data applying the Mplus package, version 7.0. To assess model fit, we used the root mean square error of approximation (RMSEA), the comparative fit index (CFI), and the standardized root mean square residual (SRMR). Values below 0.08 for RMSEA, below 0.09 for SRMR, and above 0.90 for CFI indicate acceptable model fit (Schweizer, 2010).

## Results

### Exploratory Factor Analysis on Centered Data

First, we performed an EFA on the 57 statements centered around the group mean. The determinant of the correlation matrix was close to zero, the Kaiser-Meyer-Olkin (KMO) measure of sampling adequacy was 0.93, and Bartlett’s test of sphericity was statistically significant (χ^2^ = 10247.36, *df* = 1596, *p* < 0.001). Based on Kaiser’s criterion (eigenvalues > 1.0), we identified 12 factors, which accounted for 59% of the total variance. As the results were uninterpretable, with low factor loadings and theoretically discordant items loading together on some factors, we removed some items and ran another EFA. Items were excluded based on two criteria: they were removed when their factor loadings (1) did not exceed 0.40 or (2) had cross-loadings of 0.30 on other factors. It was also expected that each factor would be represented by three items. As shown in **Table 2**, the final EFA model (KMO = 0.85, Bartlett’s test χ^2^ = 2191.81, *df* = 153, *p* < 0.001) comprised 18 items loading on six factors which accounted for 59.5% of the total variance. The first factor, called entrepreneur satisfaction, accounted for 28.6% of variance. It concerns the entrepreneur’s contentment with his or her own work, regarded as a passion and a source of his/her positive emotions. The second factor, entrepreneur work-life balance, accounted for 9.5% of variance. It captures the entrepreneur’s competent management of work and family life without bringing work-related affect home and the other way around. The third factor accounted for 8.3% of variance and was named firm social responsibility, as it captures the firm’s engagement in socially responsible activities and doing business in an ecologically responsible way. The fourth factor, accounting for 6.4% of variance, consisted of items concerning the firm’s existence on the market as well as its credibility and trustworthiness, and was called firm reputation. The fifth factor, explaining 5.9% of variance, concerned the satisfaction of the firms’ employees, their work engagement, and their positive opinions about the firm; it is called employees satisfaction. The sixth factor, called clients satisfaction, explained 5.8% of variance and captured the firm’s positive reputation among customers, their satisfaction, and their positive relations with the firm.

**Table 2:** Dimensions of entrepreneurial success as evaluated by external stakeholders: results of the exploratory factor analysis (EFA) on group mean centered data.

Indicator of entrepreneurial success	Factor loadings					
	
	1	2	3	4	5	6
(1) Running the company gives the entrepreneur a lot of satisfaction	**0.85**	-0.01	0.11	0.01	0.06	-0.02
(2) Running the company, the entrepreneur finds fulfillment at work	**0.77**	0.05	-0.00	0.02	-0.01	0.13
(3) Running the company is the entrepreneur’s passion	**0.76**	0.03	-0.07	0.04	-0.16	0.00
(4) Running the company, the entrepreneur does not carry over their professional life home	0.00	**0.74**	0.03	-0.10	-0.04	0.08
(5) Problems at work do not influence the entrepreneur’s private life	-0.05	**0.72**	0.16	0.10	-0.07	-0.08
(6) Running the company, the entrepreneur can find a balance between family life and work	0.24	**0.68**	-0.10	0.03	-0.08	0.07
(7) The company engages in charitable activities	0.13	0.03	**0.83**	0.05	0.14	-0.11
(8) The company sponsors various social initiatives	-0.07	0.12	**0.76**	0.04	-0.05	-0.01
(9) The company cares for the natural environment	-0.01	-0.05	**0.60**	-0.04	-0.16	0.16
(10) The company exists on the market long enough to inspire trust	-0.01	0.09	-0.04	**0.79**	0.01	0.02
(11) The company has experience in its sector	0.02	-0.18	0.05	**0.77**	-0.06	-0.00
(12) The time that the company has been on the market attests to its credibility	0.02	0.05	0.02	**0.74**	0.01	0.05
(13) The company’s employees recommend their employer to others	-0.10	0.02	0.02	0.05	**-0.83**	0.08
(14) Employees identify with their company	0.06	0.08	0.07	-0.02	**-0.72**	0.05
(15) The company’s employees are satisfied with their job	0.15	0.06	-0.05	0.05	**-0.72**	-0.13
(16) Customers are satisfied with the services/products provided by the company	-0.01	0.19	-0.05	0.12	0.12	**0.80**
(17) Customers recommend the company to others	0.06	-0.02	-0.02	0.01	-0.05	**0.80**
(18) The company is well-regarded by the local community	0.13	-0.21	0.18	0.02	-0.17	**0.60**


**Numbers in bold indicate strong component loadings*.*

### Multilevel Exploratory Factor Analysis

We were interested in what dimensions of entrepreneurial success would emerge on each of the two levels as well as in whether they would show the same structure at both levels. Therefore in the next step, we performed the multilevel EFA on 18 non-centered items representing evaluations done by each stakeholder of the firm. In this analysis, the number of observations on Level 1 was 590, and the number of observations on Level 2 was 57 (8.3 observations per cluster on average). The intraclass correlation coefficients (ICC) ranged from 0.12 (for the item: *Running the company, the entrepreneur does carry over their professional life home*) to 0.41 (for the following items: *The company exists on the market long enough to inspire trust; The company engages in charitable activities*). This proves that evaluations of a given firm are more similar than evaluations of different firms, which supports the choice of multilevel analysis.

The results of the two-level EFA provide insight into the structure and dimensions of entrepreneurial success on Level 1 (within) and Level 2 (between). Eigenvalues for the solution at Level 1 suggested six-factors (5.07, 1.72, 1.48, 1.14, 1.06, 1.03, 0.82, 0.74, 0.64, 0.62), while eigenvalues at Level 2 suggested a four-factors solution (8.42, 3.12, 2.06, 1.07, 0.92, 0.84, 0.52, 0.37, 0.26, 0.18). We tested a few alternative models in order to choose the best factor solution: (1) five factors at Level 1 and four factors at Level 2 (χ^2^ = 263.794, *df* = 160, *p* < 0.000, CFI = 0.959, SRMR_(within)_ = 0.033, SRMR_(between)_ = 0.068); (2) five factors at Level 1 and five factors at Level 2 (χ^2^ = 239.692, *df* = 146, *p* < 0.000, CFI = 0.963, SRMR_(within)_ = 0.033, SRMR_(between)_ = 0.048); (3) six factors at Level 1 and four factors at Level 2 (χ^2^ = 170.769, *df* = 147, *p* < 0.087, CFI = 0.991, SRMR_(within)_ = 0.021, SRMR_(between)_ = 0.066); (4) six factors at Level 1 and five factors at Level 2 (χ^2^ = 145.479, *df* = 133, *p* < 0.217, CFI = 0.995, SRMR_(within)_ = 0.021, SRMR_(between)_ = 0.047). Considering not only model fit but also interpretability, simple structure, and explained variance, the model with six factors at Level 1 and four factors at Level 2 appeared to offer the best solution.

This final model of the two-level factorial structure of entrepreneurial success is presented in **Table 3**. The six dimensions obtained at Level 1 capture the perception of the firm by each of its individual external stakeholders; they explain 63.9% of total variance. The factorial structure obtained at this level is identical to the structure obtained in EFA on the group mean centered data with the following factors: (1) entrepreneur satisfaction (28.2% of explained variance); (2) entrepreneur work-life balance (9.6% of explained variance); (3) firm social responsibility (8.2% of explained variance); (4) firm reputation (6.3% of explained variance); (5) employees satisfaction (5.9% of explained variance); (6) clients satisfaction (5.7% of explained variance).

**Table 3:** Dimensions of entrepreneurial success as evaluated by external stakeholders: results of the multilevel factor analysis (factor loadings).

Indicator	Stakeholder level (Level 1)						Firm level (Level 2)			
		
	1	2	3	4	5	6	1	2	3	4
1.	**0.68**	0.01	0.12	-0.01	-0.02	0.03	**0.82**	0.32	-0.05	-0.00
2.	**0.72**	0.03	0.02	0.01	0.02	0.09	**0.91**	0.13	0.02	0.07
3.	**0.66**	0.02	-0.06	0.04	0.16	-0.02	**1.03^∗^**	0.00	0.17	-0.01
4.	0.05	**0.51**	0.11	-0.14	0.04	0.02	-0.37	**0.86**	0.09	-0.06
5.	-0.02	**0.58**	0.18	0.05	0.03	-0.08	-0.09	**0.42**	0.35	0.03
6.	0.19	**0.68**	-0.05	0.03	0.01	0.02	0.25	**0.57**	-0.02	-0.20
7.	0.08	-0.05	**0.78**	0.00	-0.06	-0.04	-0.18	0.25	**0.77**	0.01
8.	-0.08	0.09	**0.60**	-0.00	0.10	0.04	0.05	-0.02	**1.05**	0.16
9.	-0.02	0.06	**0.35**	0.06	0.14	0.06	0.27	**0.52**	0.26	-0.01
10.	0.03	0.09	-0.02	**0.61**	-0.04	0.03	0.02	0.10	0.04	**0.90**
11.	0.01	-0.10	0.03	**0.69**	0.03	-0.03	-0.01	0.17	-0.02	**0.85**
12.	-0.01	0.05	0.02	**0.55**	0.05	0.07	0.04	-0.06	-0.06	**1.00**
13.	-0.07	-0.01	-0.02	0.02	**0.79**	0.03	-0.33	0.34	0.09	**0.48**
14.	0.11	0.06	0.05	-0.03	**0.60**	0.03	0.03	**0.96**	0.11	-0.14
15.	0.16	0.04	-0.01	0.02	**0.48**	-0.10	-0.18	**0.98**	-0.22	0.08
16.	-0.03	0.23	-0.01	0.01	-0.04	**0.77**	0.04	**0.99**	-0.18	0.04
17.	0.07	-0.02	-0.02	-0.02	0.17	**0.58**	0.12	**0.89**	-0.30	0.08
18.	0.09	-0.14	0.10	0.09	0.23	**0.40**	0.04	**0.72**	0.04	0.22


*Numbers in bold indicate strong component loadings; ^∗^the loading >1 is possible because the factors are assumed to be correlated (oblique rotation) (Harrigan et al., 2018)*.

The four dimensions of entrepreneurial success obtained at Level 2 capture the perception of the firm by all of its external stakeholders. The four factors explained 81.4% of total variance. The first factor (46.8% of explained variance) – entrepreneur satisfaction – is identical with the analogous factor obtained at Level 1, as the same items belong to this factor at both levels. The second factor (17.3% explained variance), called relations with the environment, is made up of items concerning balance between work and personal life, the firm’s social responsibility, as well as satisfaction of its employees and clients. The third factor – pro-social activity (11.4% explained variance) is made of two statements regarding the firm’s charity work and sponsoring social events. The last factor, called firm credibility (5.9% explained variance), comprises four statements representing both customers’ and employees’ trust in the firm.

### Correlations Between Entrepreneurial Success Dimensions

Descriptive statistics, correlations, and internal consistency coefficients for all dimensions of entrepreneurial success obtained at each level are presented in **Tables 4**, **5**. The score for each dimension at Level 1 was calculated as the mean value of items with high individual-level factors loadings on a given factor (see **Table 3**). At Level 2, we computed the firm average of evaluations across its stakeholders based on the items with high firm-level factor loadings. At Level 1, all success dimensions are positively correlated and all these correlations are statistically significant. At Level 2, entrepreneur satisfaction is positively and significantly correlated with relations with the environment but not with the other two dimensions. This suggests that entrepreneur satisfaction is relatively unrelated to other success dimensions on the firm level.

**Table 4:** Descriptive statistics and correlations between dimensions of entrepreneurial success at the stakeholder level (Level 1).

Dimension of entrepreneurial success	*M*	*SD*	1	2	3	4	5	6
(1) Entrepreneur satisfaction	4.33	0.70	*0.83*					
(2) Entrepreneur work-life balance	3.61	0.82	0.35^∗∗^	*0.68*				
(3) Firm social responsibility	3.12	0.97	0.20^∗∗^	0.33^∗∗^	*0.71*			
(4) Firm reputation	4.44	0.66	0.33^∗∗^	0.20^∗∗^	0.27^∗∗^	*0.81*		
(5) Employees satisfaction	4.07	0.67	0.45^∗∗^	0.42^∗∗^	0.37^∗∗^	0.42^∗∗^	*0.74*	
(6) Clients satisfaction	4.36	0.61	0.47^∗∗^	0.26^∗∗^	0.27^∗∗^	0.50^∗∗^	0.50^∗∗^	*0.77*


**^∗^p < 0.05; ^∗∗^p < 0.001 (two-tailed); Cronbach’s alpha reliability is reported on the diagonal (presented as italicized)*.*

**Table 5:** Descriptive statistics and correlations between dimensions of entrepreneurial success at the firm level (Level 2).

Dimension of entrepreneurial success	*M*	*SD*	1	2	3	4
(1) Entrepreneur satisfaction	4.33	0.38	*0.91*			
(2) Relations with the environment	4.05	0.35	0.54^∗∗^	*0.87*		
(3) Pro-social activity	2.74	0.86	-0.03	0.33^∗^	*0.92*	
(4) Firm credibility	4.35	0.44	0.25	0.58^∗∗^	0.27^∗^	*0.89*


**^∗^p < 0.05; ^∗∗^p < 0.001 (two-tailed); Cronbach’s alpha reliability is reported on the diagonal (presented as italicized)*.*

### Differences Across Firms on Entrepreneurial Success Dimensions

To check whether the evaluation of entrepreneurial success depends on firm characteristics, we tested for potential differences in each of the four success dimensions identified at Level 2 (see **Table 5**). We compared the mean level of each dimension across firms, taking two characteristics of firms into account: their size and the time of their existence on the market.

As far as size of a firm is concerned, we divided all firms (median split) into those hiring up to eight employees (*n* = 37) and those hiring more than eight employees (*n* = 20). The results show that the evaluation of any of the four entrepreneurial success dimensions does not differ significantly between these two groups: entrepreneur satisfaction (*U* = 352.00, *p* = 0.763, *M _≤_*_8_ = 4.34, *SD* = 0.38, *M*_>__8_ = 4.31, *SD* = 0.40), relations with the environment (*U* = 357.50, *p* = 0.834, *M _≤_*_8_ = 4.05, *SD* = 0.37, *M*_>__8_ = 4.04, *SD* = 0.31), pro-social activity (*U* = 358.50, *p* = 0.847, *M _≤_*_8_ = 2.76, *SD* = 0.92, *M*_>__8_ = 2.70, *SD* = 0.78), firm credibility (*U* = 368.00, *p* = 0.631, *M _≤_*_8_ = 4.35, *SD* = 0.43, *M*_>__8_ = 4.35, *SD* = 0.47). This demonstrates that the size of a micro-firm is not related to whether it is perceived by external stakeholders as more or less successful.

Next, we checked whether the length of the firm’s existence was related to the evaluation of its success. We compared firms operating on the market for up to 14 years (*n* = 30) and for more than 14 years (*n* = 27; median split). The results revealed that there were no statistically significant differences between these two groups of firms in the evaluation of entrepreneur satisfaction (*U* = 405.00, *p* = 1.00, *M _≤_*_14_ = 4.32, *SD* = 0.42, *M*_>__14_ = 4.34, *SD* = 0.35) and relations with the environment (*U* = 323.00, *p* = 0.190, *M _≤_*_14_ = 3.98, *SD* = 0.40, *M*_>__14_ = 4.13 *SD* = 0.27). We found out, however, that firms operating for up to 14 years are rated lower on pro-social activity dimensions (*U* = 265.00, *p* = 0.025, effect size *r* = 0.30, *M _≤_*_14_ = 2.51, *SD* = 0.73, *M*_>__14_ = 3.00, *SD* = 0.93) and lower on firm credibility (*U* = 220.50, *p* = 0.003, effect size *r* = 0.39, *M _≤_*_14_ = 4.17, *SD* = 0.51, *M*_>__14_ = 4.55, *SD* = 0.23) in comparison with firms existing longer on the market. This means that stakeholders’ evaluation of entrepreneurial success on these two dimensions depends on how long a given firm has existed, with more mature firms being appreciated.

## Discussion

The aim of the study was to identify the dimensions of entrepreneurial success of micro-firms as seen from the external perspective of their stakeholders – business partners. The results of the EFA on centered data showed that stakeholders perceive entrepreneurial success using six dimensions: (1) entrepreneur satisfaction; (2) entrepreneur work-life balance; (3) firm social responsibility; (4) firm reputation; (5) employees satisfaction; (6) clients satisfaction. Thanks to applying two-level EFA we were able to verify the structure of entrepreneurial success taking into account its variance on two levels simultaneously: at the individual stakeholder level (Level 1) and at the firm level (Level 2). The results of the multilevel factor analysis confirmed the six dimensions of entrepreneurial success listed above at the level of individual stakeholders. They are similar to those obtained in earlier studies based on evaluations made by entrepreneurs (Gorgievski et al., 2011; Dijkhuizen et al., 2016; Przepiorka, 2016; Wach et al., 2016). The analysis of these dimensions may be of interest in future psychological studies, which may, for example investigate individual antecedents of entrepreneurial success as perceived by external business partners. At the firm level we found four success dimensions allowing for the evaluation of the firm as a whole: (1) entrepreneur satisfaction; (2) relations with the environment; (3) pro-social activity; (4) firm credibility. They may be of interest in further studies, using a macro perspective and looking at firms rather than at individual stakeholders, which may be interesting for economic or organizational studies. What is worth noting is that no distinct dimension concerning the economic or financial results of the firm emerged in evaluations of entrepreneurial success made by stakeholders. These results are consistent with the findings of previous studies (Greenbank, 2001; Walker and Brown, 2004; Reijonen and Komppula, 2007), suggesting that financial indicators not always reflect the success of micro-enterprises accurately.

The results of our analysis suggest that the dimensions of entrepreneurial success used in evaluations by individual stakeholders and the dimensions used at the firm level are not the same. Therefore, there is no construct isomorphism – i.e., no structural equivalence of entrepreneurial success across different levels of analysis (Tay et al., 2014; Alessandri et al., 2017). Such isomorphism is observed when at least the same number of factors exist at both levels of analysis. Thus, it seems that at the firm level the meaning of entrepreneurial success is quite different from its meaning at the individual stakeholder level. This result should be subject to further investigation in future studies.

The results of the initial qualitative study are also worth noting. Even though its purpose was to identify indicators of entrepreneurial success as seen by external stakeholders, it also brings interesting observations. Stakeholders confirmed that the indicators of entrepreneurial success identified in earlier research on entrepreneurs (Buttner and Moore, 1997; Gorgievski et al., 2011, 2014; Fisher et al., 2014) were also noteworthy for them. Describing the success of the firm they cooperate with, they pay attention to its image, which is expressed, for example, by customer service standards, positive associations created by advertising, but also the esthetic features of a firm, conveniences such as a car park or elevator. Other indicators of entrepreneurial success listed in the literature (van Praag and Versloot, 2007; Richard et al., 2009), such as the financial performance of the firm or its competitiveness, are also taken into account by external partners. What was not obvious, however, is the presence of employee satisfaction as an entrepreneurial success criterion important for external stakeholders. This shows that, for external partners, positive opinions of micro-firms’ employees, their work engagement, and lack of turn-over intention, are vital indicators that the firm is prospering well and thriving on the market. This leads to the conclusion that for micro-firms it is worth caring about employees, who are the flagship of a company for its external stakeholders.

Thanks to the multilevel study design, which is as yet rare in entrepreneurship research (Laguna et al., 2016), our study offers a more nuanced understanding of the different faces of entrepreneurial success. Our conceptualization affords a look at the entrepreneurial success from the perspective of people who cooperate with micro-firms on a daily basis. It depends on their evaluations of the company’s prosperity whether they keep the collaboration going or not (Freeman, 1984). Such conceptualization of entrepreneurial success has been rare in the literature to date. Thanks to the present study we know which categories and at which level are used to evaluate entrepreneurial success, which brings us closer to a better understanding of the complex relations between entrepreneurs, their companies, and the environment. Since the prosperity of firms, especially of micro- and small enterprises, has profound social importance (e.g., van Praag and Versloot, 2007), studies on entrepreneurial success should be continued.

Although it was not the main aim of our research, the dimensions of entrepreneurial success obtained in this study may serve as the basis for the development of a tool measuring entrepreneurial success from the external perspective. Such a tool would make it possible to measure entrepreneurial success as seen by stakeholders at two levels, based on the specific indicators of success extracted from the interviews with business partners. In this measure the meaning of stakeholders’ individual-level evaluation should be based on the items with high individual-level factor loadings. At the firm level, by contrast, it can be considered as the firm average of evaluations across its stakeholders, based on the items with high firm-level factor loadings. The final development of such a measure, however, demands further studies on the validity of its scales. The results of reliability analysis presented in **Tables 4**, **5** (Cronbach’s alpha coefficients ranging from 0.68 to 0.83 at the individual level and from 0.87 to 0.92 at the firm level) confirm acceptable to high internal consistency of all scales. This kind of measure may be useful in future research. In most of the previous studies entrepreneurial success was evaluated by entrepreneurs themselves using self-assessment tools (e.g., Murphy et al., 1996; Dijkhuizen et al., 2016; Przepiorka, 2016; Wach et al., 2016). Self-evaluation, however, is prone to social approval, and when used together with other self-assessment tools measuring other constructs it may involve a risk of common method bias (Podsakoff et al., 2012). Conceptualizing entrepreneurial success from the perspective of stakeholders may prevent such bias in future studies.

### Limitations and Future Directions

Our sample brings some limitations to the research findings. First, we focused on micro-firms employing up to 10 people only. The evaluation of the success of bigger firms may involve other indicators and dimensions (e.g., presence on the stock market), which are not always appropriate for micro-firms. Second, the stakeholders taking part in our study evaluated the success of firms which had operated for at least 2 years, being relatively mature businesses. As the evaluation of entrepreneurial success may be dependent on the stage of firm development (Witt, 2004; Baron, 2007), different success dimensions may be important in the case of start-ups. Although we tested differences in stakeholders’ evaluations of entrepreneurial success between different micro-firms, further studies are needed to test the dimensions of entrepreneurial success in bigger companies and in firms operating for less than 2 years.

### Implications for Practice

Our results imply certain recommendations for the practice of micro-firm management and for those who support entrepreneurs. They suggest that, as in the case of evaluations done by entrepreneurs themselves, the evaluation of entrepreneurial success by external partners does not necessarily focus on financial outcomes (Buttner and Moore, 1997; Gorgievski et al., 2011, 2014; Fisher et al., 2014; Wach et al., 2016). For individual business partners, the important dimensions of entrepreneurial success are: good opinions about the firm, satisfaction of the entrepreneur and his/her employees, customer satisfaction, good work-life balance, and (to a lesser extent) the firm’s engagement in the local community. As regards the evaluation of the firm as a whole, it is important for a company to be trustworthy and for the entrepreneur to derive satisfaction from running it; it is also important for the firm to maintain good relations with the environment and (to a lesser extent) to engage in pro-social activities. Entrepreneurs should be made aware of the significance of these non-financial indicators of entrepreneurial success. The awareness of which dimensions a firm might be judged on by stakeholders could contribute to effective image management. In micro-firms, which usually do not have specialized marketing departments, it is the entrepreneur and his/her team who personally build the firm’s social image. Our results could also be applied for the development of professional support for entrepreneurs, such as trainings and consulting services, providing knowledge on the dimensions and indicators of entrepreneurial success as seen by business partners.

## Ethics Statement

Compliance with Ethical Standards: All procedures performed in this study were in accordance with the ethical standards. Informed consent was obtained from all individual participants included in the study. Participation in the study was voluntary and the participants did not receive any reward. Respondents were asked to fill in a set of questionnaires. They were able to withdraw from the study at each moment. The confidentiality and anonymity were ensured. The study received the approval from the Ethical Committee of The John Paul II Catholic University of Lublin, Institute of Psychology.

## Author Contributions

WR and ML were involved in formulating the research question, designing the studies, collecting and analyzing the data, writing the article, and drafting and approving the final manuscript.

## Conflict of Interest Statement

The authors declare that the research was conducted in the absence of any commercial or financial relationships that could be construed as a potential conflict of interest.
